# GrcC1 mediates low-level resistance to multiple drugs in *M. marinum*, *M. abscessus,* and *M. smegmatis*

**DOI:** 10.1128/spectrum.02289-24

**Published:** 2025-02-26

**Authors:** Cuiting Fang, Han Zhang, Jing He, Xirong Tian, Sanshan Zeng, Xingli Han, Shuai Wang, Buhari Yusuf, Jinxing Hu, Nanshan Zhong, Yamin Gao, H. M. Adnan Hameed, Tianyu Zhang

**Affiliations:** 1 State Key Laboratory of Respiratory Disease, Institute of Drug Discovery, Guangzhou Institutes of Biomedicine and Health, Chinese Academy of Sciences, Guangzhou, Guangdong, China; 2 Guangdong-HongKong-Macau Joint Laboratory of Respiratory Infectious Diseases, Guangzhou Institutes of Biomedicine and Health, Chinese Academy of Sciences, Guangzhou, Guangdong, China; 3 University of Chinese Academy of Sciences, Beijing, China; 4 China-New Zealand Belt and Road Joint Laboratory on Biomedicine and Health, Guangzhou Institutes of Biomedicine and Health, Chinese Academy of Sciences, Guangzhou, Guangdong, China; 5 School of Life Sciences, University of Science and Technology of China, Hefei, Anhui, China; 6 Institute of Physical Science and Information Technology, Anhui University, Hefei, Anhui, China; 7 State Key Laboratory of Respiratory Disease, Guangzhou Chest Hospital, Guangzhou, Guangdong, China; 8 Guangzhou National Laboratory, Guangzhou, Guangdong, China; University of Nebraska Medical Center, Omaha, Nebraska, USA

**Keywords:** GrcC1, *Mycobacterium marinum*, *Mycobacterium abscessus*, *Mycobacterium smegmatis*, drug resistance mechanism

## Abstract

The escalating threat of mycobacterial infectious diseases, particularly those caused by nontuberculous mycobacteria (NTM), poses a serious challenge to public health. Linezolid (LZD), an oxazolidinone antimicrobial, exhibits potent activity against *Mycobacterium tuberculosis* and NTM. Generally, mutations in the *rrl* and *rplC* genes are widely associated with resistance to LZD. However, in this study, we screened *Mycobacterium marinum* strains lacking such mutations, indicating the presence of an alternative resistance mechanism. Notably, through whole-genome sequencing, we identified a novel mutation C395T in the *MMAR_0911* (*grcC1*) gene that has never been linked to drug resistance. This mutation leads to an A132V substitution in the encoded protein, a polyprenyl diphosphate synthase potentially involved in the synthesis of cell wall components and menaquinones. We found that the overexpression of *grcC1* caused resistance to multiple drugs including LZD, clarithromycin (CLR), vancomycin (VAN), clofazimine (CFZ), rifampicin (RIF), cefoxitin (CEF), levofloxacin (LEV), and moxifloxacin (MXF) and reduced cell wall permeability, while the silence and knockout of *grcC1* showed increased cell wall permeability and susceptibility to these drugs. Using CRISPR/Cpf1-assisted gene editing, we confirmed that the A132V mutation conferred low-level resistance to the aforementioned drugs in *Mycobacterium abscessus* and *Mycobacterium smegmatis*. Furthermore, thin-layer chromatography analysis indicated reduced glycolipid polarity in the *grcC1* mutant strains, suggesting an impact on the cell envelope integrity. Our findings suggest that GrcC1 contributes to low-level drug resistance in mycobacteria by potentially reducing cell wall permeability, highlighting its potential as a novel target for antimicrobial agents and as a diagnostic marker.

**IMPORTANCE:**

Our study uncovers a novel drug resistance mechanism in mycobacteria, focusing on the previously uncharacterized *grcC1* gene. We identified a new mutation, A132V, in GrcC1, which is involved in cell wall component synthesis and menaquinone production. This mutation contributes to low-level resistance not only to linezolid but also to a broad range of drugs, including clarithromycin, vancomycin, and rifampicin. Through advanced techniques like CRISPR interference and gene editing, we demonstrated that GrcC1 plays a critical role in drug susceptibility and cell wall permeability across multiple *Mycobacterium* species. These findings represent the first connection between GrcC1 and drug resistance, offering new insights into combating infections caused by nontuberculous mycobacteria (NTM). Our work highlights the potential of GrcC1 as a target for novel therapeutic approaches and as a diagnostic marker for drug-resistant NTM infections.

## INTRODUCTION

Nontuberculous mycobacteria (NTM) infections are emerging as significant global public health threats, with their intrinsic resistance to numerous antimicrobial agents posing substantial challenges (1, 2). The effective treatment strategies are further complicated by the advent of acquired drug resistance, and genetic mechanisms underlying this resistance are not yet fully understood, particularly those involving undetected mutations in new genes or outside the known drug targets (3). These mutations often result in clinically significant low-to-intermediate levels of resistance (4–6). To enhance the treatment efficacy, understanding these mechanisms is essential for devising new strategies to counter resistance and for identifying new drug targets as well as diagnostic resistance markers.

Linezolid (LZD), an oxazolidinone, is not only a key component of the essential treatment regimen for drug-resistant tuberculosis (TB) but also widely used for treating NTM-related infections (7, 8). LZD exerts its antibacterial activity by inhibiting the protein synthesis at the ribosome level; it binds to the peptidyl transferase center of the ribosome, preventing the formation of a functional 70S initiation complex required for bacterial protein synthesis (9). Most resistance mechanisms in *Mycobacterium tuberculosis* and NTM against LZD that have been documented thus far entail alterations of LZD-binding sites, including mutations in 23S rRNA and ribosomal proteins (L3, L4, and L22), or modifications of 23S rRNA (10, 11). Recently, only 8.2% of LZD-resistant *Mycobacterium abscessus* isolates were found to have 23S rRNA mutations, whereas 19.8% of LZD-resistant *M. tuberculosis* isolates lacked mutations in known resistance genes, indicating the existence of alternative and unexplored resistance mechanisms (11, 12).

In this study, we identified nine LZD-resistant *Mycobacterium marinum* strains that lacked mutations in *rplC* and *rrl*. For the first time, whole-genome sequencing (WGS) revealed a novel mutation (C395T) in the *grcC1* gene, which encodes geranylgeranyl diphosphate (GGPP) synthase, an enzyme responsible for synthesizing GGPP—a 20-carbon polyprenyl phosphate (Pol-P) lipid involved in respiration and maintaining cell membrane structure (13, 14). Through overexpression, CRISPR interference (CRISPRi), and CRISPR/Cpf1-assisted gene knockout, we demonstrated that the expression level of *grcC1* influences the susceptibility of mycobacteria to multiple drugs, including LZD, clarithromycin (CLR), vancomycin (VAN), clofazimine (CFZ), rifampicin (RIF), cefoxitin (CEF), levofloxacin (LEV), and moxifloxacin (MXF) by affecting cell wall permeability. Moreover, using CRISPR/Cpf1-assisted gene editing, we confirmed that A132V mutation in the GrcC1 leads to low-level resistance to aforesaid drugs in *Mycobacterium smegmatis* and *M. abscessus*. Our novel findings underscore that GrcC1 could serve as a potential therapeutic drug target for developing new antimicrobial agents and a potential diagnostic marker for drug-resistant NTM infections.

## RESULTS

### Cross-resistance of LZD-resistant *M*. *marinum* strains to multiple drugs lacking *rrl* and *rplc* mutations

To explore alternative mechanisms of LZD resistance, we used the potential model mycobacteria of *M. tuberculosis* such as *M. marinum* and *M. smegmatis*, both of which are sensitive to LZD, with a minimal inhibitory concentration (MIC) of 2 µg/mL (15, 16). These mycobacterial models provide advantages over *M. tuberculosis* by offering faster growth rates and the availability of genetic tools (17). *M. abscessus*, a rapidly growing mycobacterium often implicated in severe human infections and widely known for its intrinsic drug-resistant nature, was also included to provide valuable insights into LZD resistance mechanisms (2, 18). Given the intrinsic high-level resistance of *M. abscessus* GZ002 (Mab^WT^) to LZD (MIC = 64 µg/mL), we used the *embC* gene knock-out strain of Mab^WT^ (Mab^ΔembC^), which is sensitive to LZD (MIC = 2 µg/mL), to screen for LZD-resistant strains (19, 20). Our initial screening encompassed all three NTM species for spontaneous LZD-resistant strains, which were subsequently subjected to Sanger sequencing of the *rplC* and *rrl* genes.

During the screening of *M. smegmatis* mc^2^155 (Msm^WT^) mutants, Sanger sequencing revealed that the G1068A mutation predominantly occurred in the *rrlA* gene. Simultaneously, we identified 15 (6.7%, 15/224) low-level LZD-resistant Mab^ΔembC^ strains that lacked mutations in *rplC* and *rrl*. Based on a prior study, we suspected these strains might harbor compensatory mutations in *embB* (21). Sanger sequencing confirmed that all of these Mab^ΔembC^ strains had a G916A mutation in *embB*, potentially contributing to LZD resistance. Consequently, no further investigation was conducted on these resistant mutants.

We ultimately acquired nine (18.37%, 9/49) LZD-resistant autoluminescent *M. marinum* (AlMmr) strains lacking mutations in *rplC* and *rrl* genes (Table 1) (22). These strains exhibited a moderate level of resistance to LZD, with a fourfold increase in the MICs. Additionally, we performed drug susceptibility testing (DST) on these AlMmr strains to other antimicrobial agents commonly used for treating AlMmr infection (15). Interestingly, the strains showed increased resistance to RIF, CLR, VAN, and MXF, with MICs elevated by twofold to eightfold. Notably, the MICs of VAN increased up to 16-fold against some strains. These findings suggest that the resistance mechanisms in these LZD-resistant strains may not be specific to LZD and could potentially lead to cross-resistance to multiple drugs.

**TABLE 1:** MICs of LZD, RIF, CLR, MXF, and VAN against LZD-resistant AlMmr strains without mutation in *rplC* and *rrl* genes

Strain no.	MICs (μg/mL)^*a*^				
	LZD	RIF	CLR	MXF	VAN
AlMmr	2	0.125	1	0.5	0.25
AlMmr-R1-1	8	0.25	4	1	4
AlMmr-R1-3	8	0.125	2	1	8
AlMmr-R1-4	8	0.25	2	2	8
AlMmr-R1-5	8	0.25	2	2	8
AlMmr-R1-15	8	0.5	4	4	4
AlMmr-R1-16	16	0.5	4	2	4
AlMmr-R4-1	8	0.25	2	1	4
AlMmr-R4-4	8	0.25	2	4	8
AlMmr-R4-12	8	0.5	4	2	4

^*a*^ MICs against AlMmr were obtained by detecting the relative light units (RLUs) (22).

### WGS of AlMmr mutants identified novel mutations in *grcC1* and *MMAR_2832*, potentially associated with drug resistance

To elucidate the unexplored resistance mechanisms, AlMmr strains were investigated through WGS analysis, and five mutations were identified across four genes (Table 2). The most prevalent mutations were C395T in *MMAR_0911* (*grcC1*) and T1708A in *MMAR_2832*, both of which were present in all nine mutants. These findings imply that these mutations may play a role in the observed LZD resistance in our mutant strains.

**TABLE 2:** Mutations identified through WGS analysis of AlMmr LZD-resistant mutants

Gene	Product	Functional category	Mutation (nucleotide/ amino acids)	Number of mutants with mutation
*MMAR_0911* (*grcC1*)	Polyprenyl diphosphate synthetase	Intermediary metabolism and respiration	C395T / A132V	9
*MMAR_2832*	Conserved hypothetical protein	Unknown	T1708A / F570I	9
*MMAR_4284*	Transcriptional regulatory protein	Regulatory proteins	588insC^*a*^ / K197fs^*b*^	1
			A113G / Y38C	1
*MMAR_1594*	PE-PGRS family protein	Pe/ppe	G9628A / A3210T	1

^*a*^ ins: insertion.

^*b*^ fs, frameshift.

**Fig 1:**
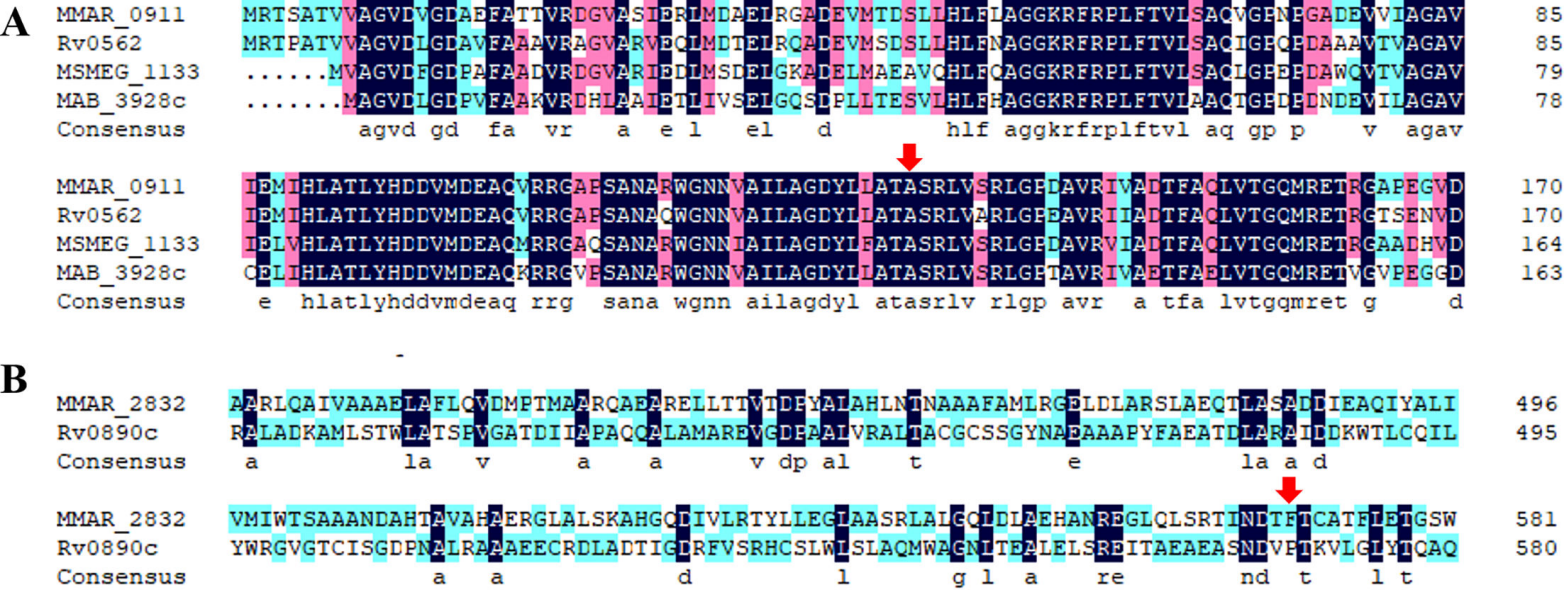
Alignments of partial amino acid sequences of GrcC1 (**A**) and MMAR_2832 (**B**) across *M. tuberculosis*, *M. marinum*, *M. smegmatis*, and *M. abscessus*. Red arrows indicate the mutation sites found in the LZD-resistant AlMmr mutants.

Through BLAST and sequence alignment, we found that GrcC1 is highly conserved among *M. marinum* (MMAR_0911), *M. tuberculosis* (Rv0562), *M. smegmatis* (MSMEG_1133), *and M. abscessus* (MAB_3928c), sharing 82.76% identity in amino acid sequences (Fig. 1A). Moreover, the A132 site in MMAR_0911 is conserved, indicating that investigating this mutation in other mycobacterial species might be feasible. In contrast, BLAST analysis showed that MMAR_2832 shares 21.69% identity with Rv0890c, a LuxR family transcriptional regulator (Fig. 1B). However, MMAR_2832 lacks a homologous protein in other rapidly growing mycobacterial strains such as *M. smegmatis* and *M. abscessus*.

### *grcC1* overexpression in Msm^WT^ and Mab^ΔembC^ induced resistance to different drugs

To validate the roles of *grcC1* and *MMAR_2832* in LZD resistance, we inserted the wild-type (*grcC1*^WT^ and *MMAR_2832*^WT^) and mutant versions of these genes (*grcC1*^A132V^ and *MMAR_2832*^F570I^), as well as homologous genes from *M. tuberculosis*, *M. smegmatis*, and *M. abscessus*, into the extrachromosomal plasmid pMV261A.

**Fig 2:**
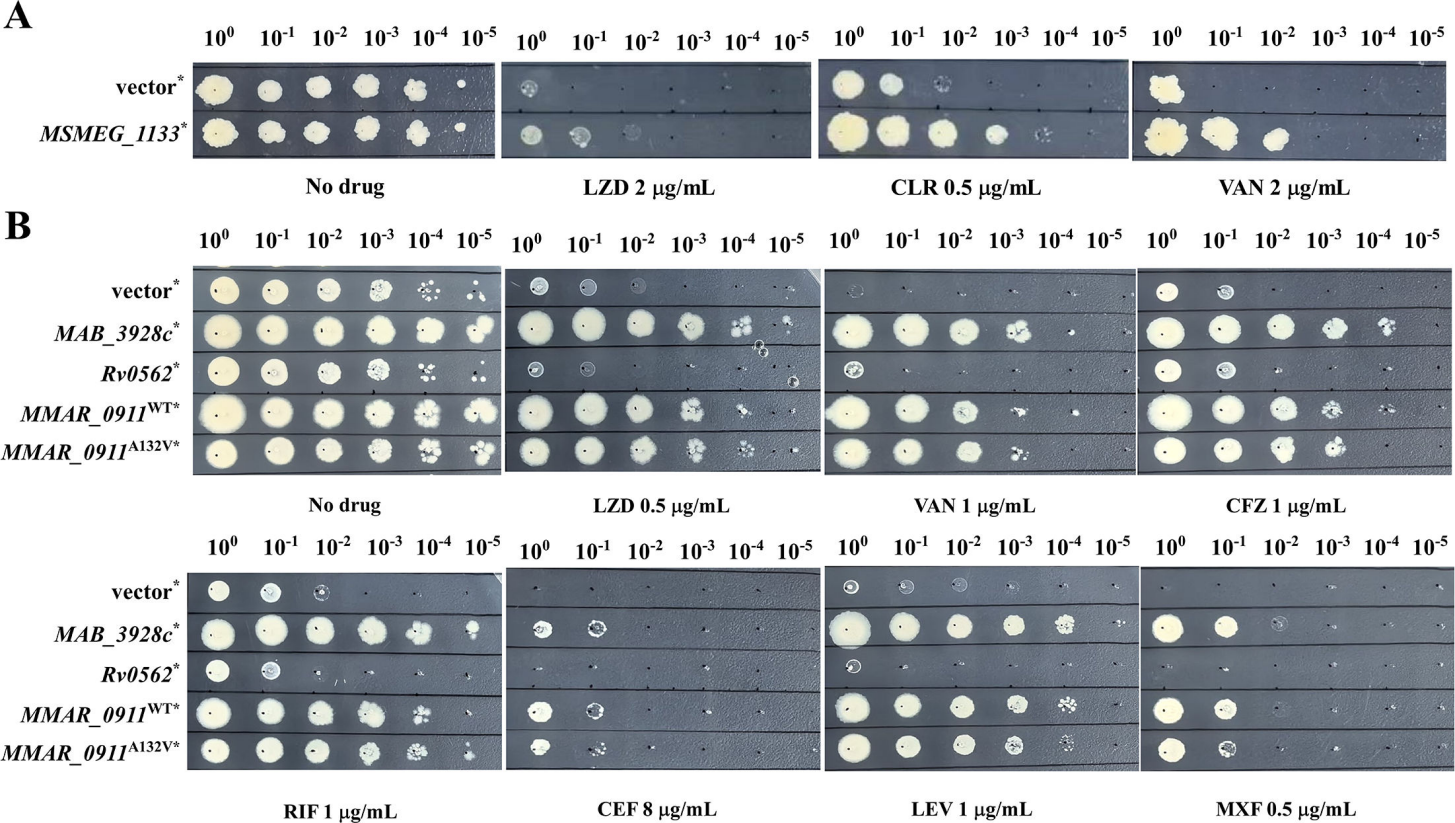
Overexpression of *grcC1* decreased drug sensitivities of Msm^WT^ and Mab^ΔembC^ to LZD, CLR, VAN, CFZ, RIF, CEF, LEV, and MXF. (**A**) The sensitivity of Msm^WT^ to LZD, CLR, and VAN upon *grcC1* overexpression. (**B**) The sensitivity of Mab^ΔembC^ to LZD, VAN, CFZ, RIF, CEF, LEV, and MXF upon *grcC1* overexpression. ^*^, strains harboring pMV261A-based plasmids, inserted with *MSMEG_1133*, *MAB_3928*c, *Rv0562*, *MMAR_0911*^WT^, and *MMAR_0911*^A132V^; vector, pMV261A.

We observed that the overexpression of *MAB_3928*c and *MSMEG_1133* resulted in a fourfold increase in the MICs of LZD in Mab^ΔembC^ and Msm^WT^ separately (Table 3). Additionally, the overexpression of *MSMEG_1133* in Msm^WT^ also conferred resistance to CLR and VAN (Fig. 2A). In Mab^ΔembC^, the overexpression of *MAB_3928* c led to resistance against a broader range of drugs, including LZD, VAN, CFZ, RIF, CEF, LEV, and MXF (Fig. 2B). Interestingly, the overexpression of *grcC1* from *M. marinum* had divergent effects on Msm^WT^ and Mab^ΔembC^ as it decreased the susceptibility of Mab^ΔembC^ to multiple drugs including LZD, VAN, CFZ, RIF, CEF, LEV, and MXF; however, it did not affect the susceptibility of Msm^WT^ to these drugs. This indicates that *grcC1* overexpression influences the drug susceptibility to multiple antimicrobial agents, with its impact varying significantly among mycobacterial strains.

**TABLE 3:** MICs of LZD against *grcC1* overexpressing strains

Strains	MICs (μg/mL)
Mab^ΔembC^	1
Mab^ΔembC^::*MAB_3928*c	4
Msm^WT^	2
Msm^WT^::*MSMEG_1133*	4

### CRISPRi-assisted *grcC1* gene silencing caused growth defects and increased susceptibility to multiple drugs in Msm^WT^, Mab^ΔembC^, and *M*. *marinum*

**Fig 3:**
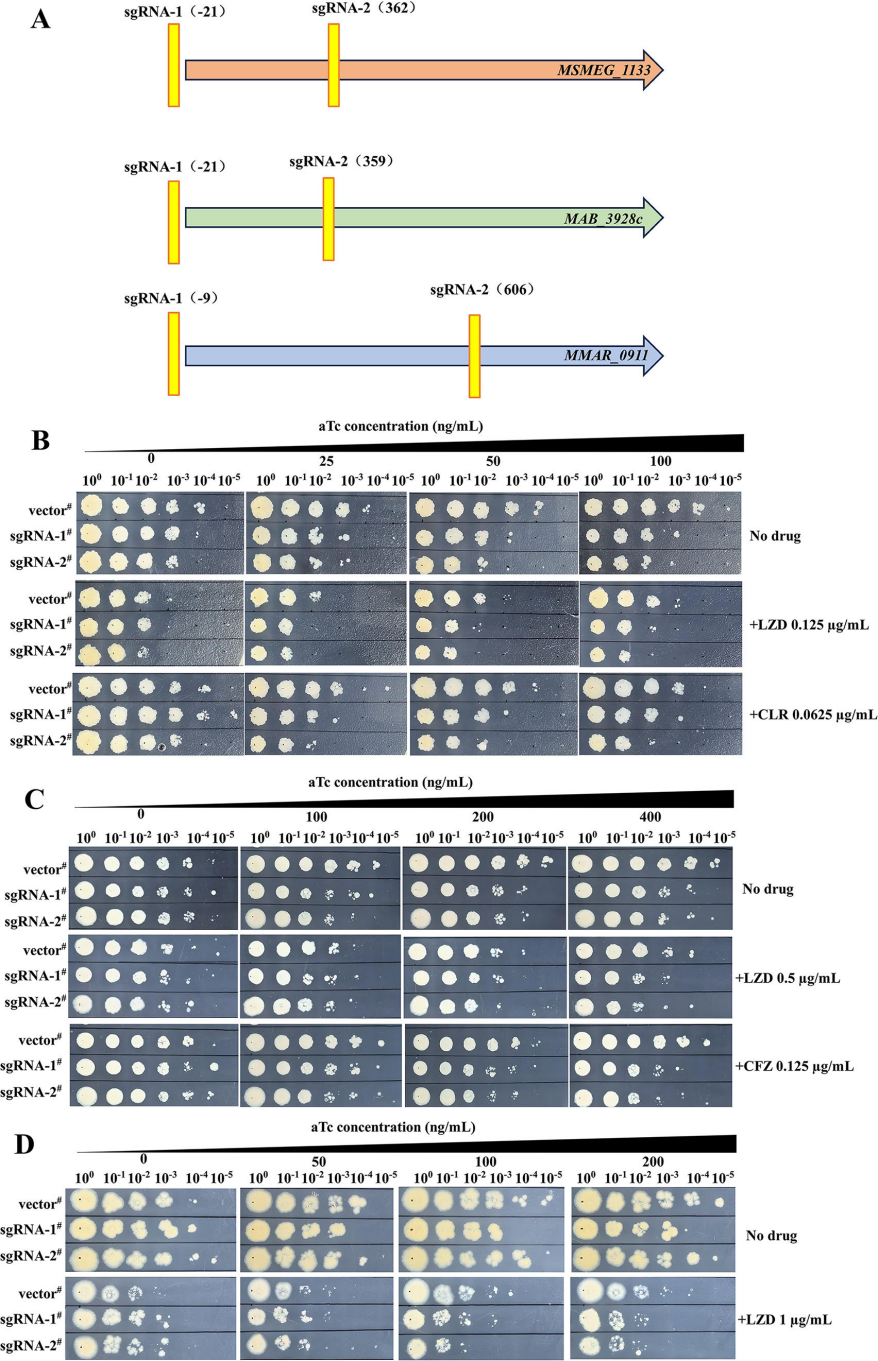
*grcC1* gene silencing inhibited growth and increased drug sensitivities in Msm^WT^, Mab^ΔembC^, and *M. marinum.* (**A**) Schematic diagram of the location of sgRNAs designed for silencing *grcC1* in different NTM species. The numbers in the brackets represent the midpoint positions of the sgRNAs within the gene. (**B**) The sensitivity of Msm^WT^ to LZD or CLR upon gene silencing. (**C**) The sensitivity of Mab^ΔembC^ to LZD or CFZ upon gene silencing. (**D**) The sensitivity of *M. marinum* to LZD upon gene silencing. ^#^, strains harboring pLJR962-based plasmids; vector, pLJR962; sgRNA-, pLJR962 inserted with corresponding sgRNA for different mycobacteria.

The varied drug susceptibility profiles observed following *grcC1* overexpression in different NTM species suggest species-specific differences in *grcC1* essentiality. While *grcC1* is annotated as an essential gene in *M. tuberculosis*, its essentiality in NTM species remains unclear. To investigate this, we employed a dCas9^sth1^-based CRISPRi system to determine the essentiality of *grcC1* and its impact on drug sensitivity in Msm^WT^, Mab^WT^, Mab^ΔembC^, and *M. marinum* (23). Two specific sgRNAs were designed for each strain (Fig. 3A). Upon silencing the *grcC1* in Msm^WT^, the highest growth dilution of the gene-silenced strains was found to be tenfold ~ hundred fold lower compared to the empty vector control, both on 7H11 agar plates with and without LZD or CLR supplementation (Fig. 3B). However, silencing *grcC1* in Mab^WT^ did not replicate the growth inhibition observed in Msm^WT^, implying that *grcC1* might not be as crucial for Mab^WT^ survival (Fig. S2). Interestingly, *grcC1* gene silencing in Mab^ΔembC^ significantly inhibited growth and increased drug sensitivities, suggesting that *grcC1* becomes more essential after the deletion of *embC* (Fig. 3C). Similarly, growth inhibition and increased sensitivity to LZD were observed in *M. marinum* following *grcC1* silencing (Fig. 3D). Conclusively, in Msm^WT^ and *M. marinum*, *grcC1* appears crucial for survival and mediates resistance to multiple drugs, while in Mab^WT^, its role is less pronounced. However, the deletion of *embC* in Mab^WT^ renders *grcC1* essential, suggesting a compensatory relationship between these two genes.

### *grcC1* knockout increased sensitivities to multiple drugs and caused growth defects in Mab^WT^

**Fig 4:**
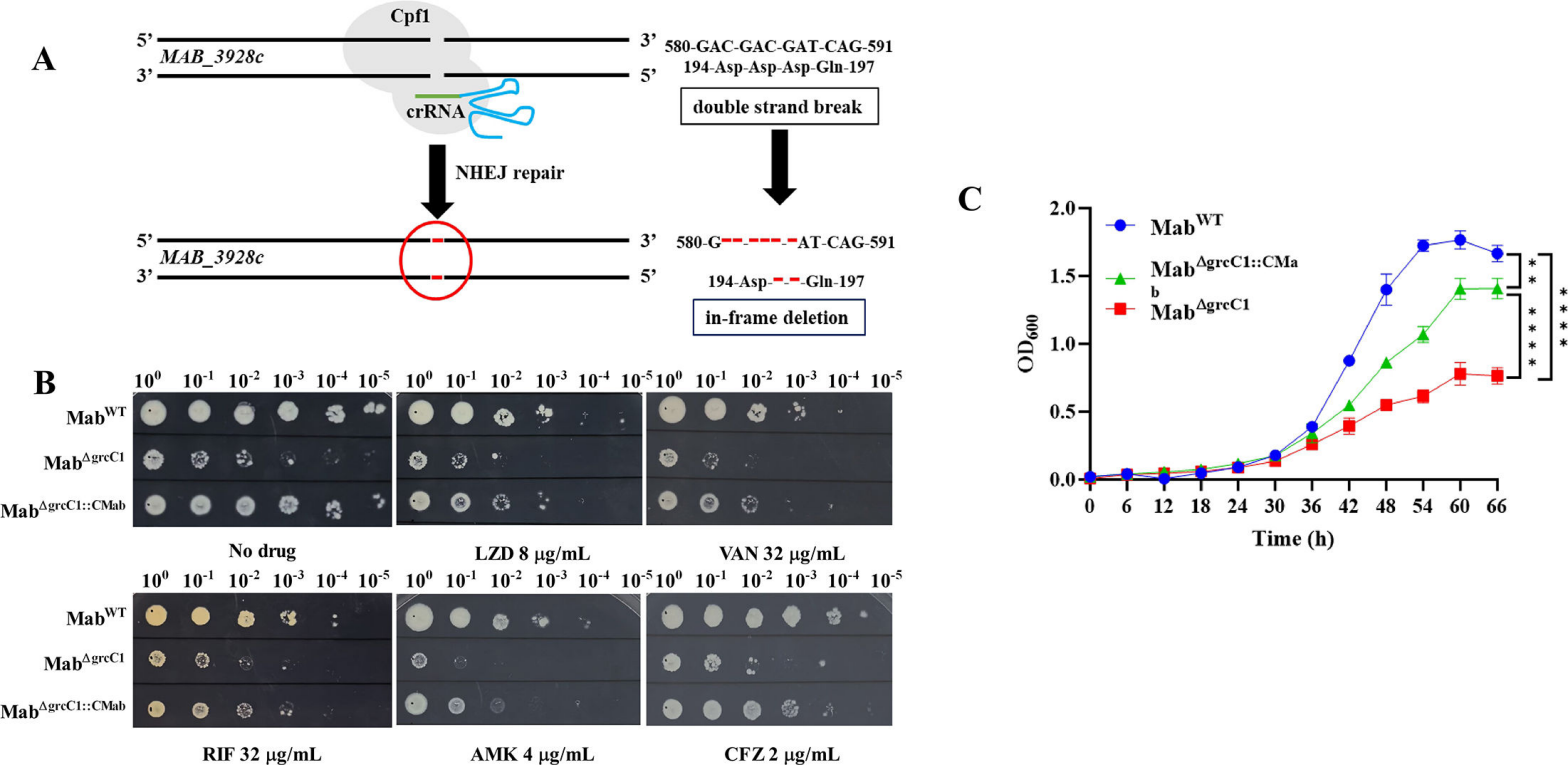
Disruption of *grcC1* increased drug sensitivities and caused growth defects in Mab^WT^. (**A**) Schematic diagram of the *grcC1* knockout in Mab^WT^. The red bars represent deleted bases and amino acids. (**B**) Sensitivity of Mab^ΔgrcC1^ and Mab^ΔgrcC1::CMab^ to LZD, VAN, RIF, AMK, and CFZ. Mab^ΔgrcC1::CMab^ refers to the complement strain. (**C**) Growth curves for Mab^WT^, Mab^ΔgrcC1^, and Mab^ΔgrcC1::CMab^. Data are presented as the mean ± standard deviation (SD), derived from a sample size of 3. Statistical significance was assessed using the area under the curve (AUC) and one-way ANOVA, where ^**^ indicates *P* < 0.01 and ^****^ indicates *P* < 0.0001.

According to our results from the gene-silencing experiments, we hypothesized that *grcC1* could be knocked out in Mab^WT^. We successfully generated a mutant (Mab^ΔgrcC1^) with a 6-bp in-frame deletion between loci 582 and 587 using the CRISPR/Cpf1-assisted nonhomologous end-joining (NHEJ) gene editing method (Fig. 4A) (24). We then performed DST on Mab^ΔgrcC1^ and the complemented strain by spotting serial dilutions of the bacterium on plates containing various drugs, including LZD, VAN, RIF, CFZ, MXF, and amikacin (AMK) (Fig. 4B). Mab^ΔgrcC1^ exhibited increased susceptibility to these drugs, while complementation with Mab-*grcC1* in Mab^ΔgrcC1^ partially restored the drug-resistant phenotype. Further investigation of the growth curves for Mab^ΔgrcC1^ and the complement strains revealed that the 6-bp deletion significantly impaired the growth rate of Mab, while complementation partially restored its growth vigor (Fig. 4C). Notably, despite the deletion not being near the N-terminal, similar to the effects observed with sgRNA-2 in *M. marinum*, disruption of *grcC1* near this region appears to impair its function (Fig. 3A and D). These findings confirm our hypothesis that *grcC1* can be disrupted in Mab^WT^. Using a more robust gene knockout approach compared to gene silencing, we demonstrated that the effects of *grcC1* knockout in Mab^WT^ are consistent with those observed in other strains through gene silencing, reinforcing the critical role of *grcC1* in maintaining drug resistance and growth across different mycobacterial species.

### Introducing *grcC1*^A132V^ into Msm^WT^ and Mab^ΔembC^ via gene editing confirmed its role in multidrug resistance

Given the conserved nature of the A132 site in GrcC1 across different mycobacterial species (Fig. 1A) and the previously reported challenges with CRISPR/Cpf1-assisted recombineering in *M. marinum* and *M. tuberculosis*, we introduced the homologous mutations of *grcC1*^A132V^ into Msm^WT^ (A126V) and Mab^ΔembC^ (A125V) to further elucidate the role of this novel mutation (25, 26). Additionally, we generated an edited *M. smegmatis* strain with the L117M mutation in GrcC1, homologous to the L122M mutation reported in an extensive drug-resistant *M. tuberculosis* clinical isolate (27).

**Fig 5:**
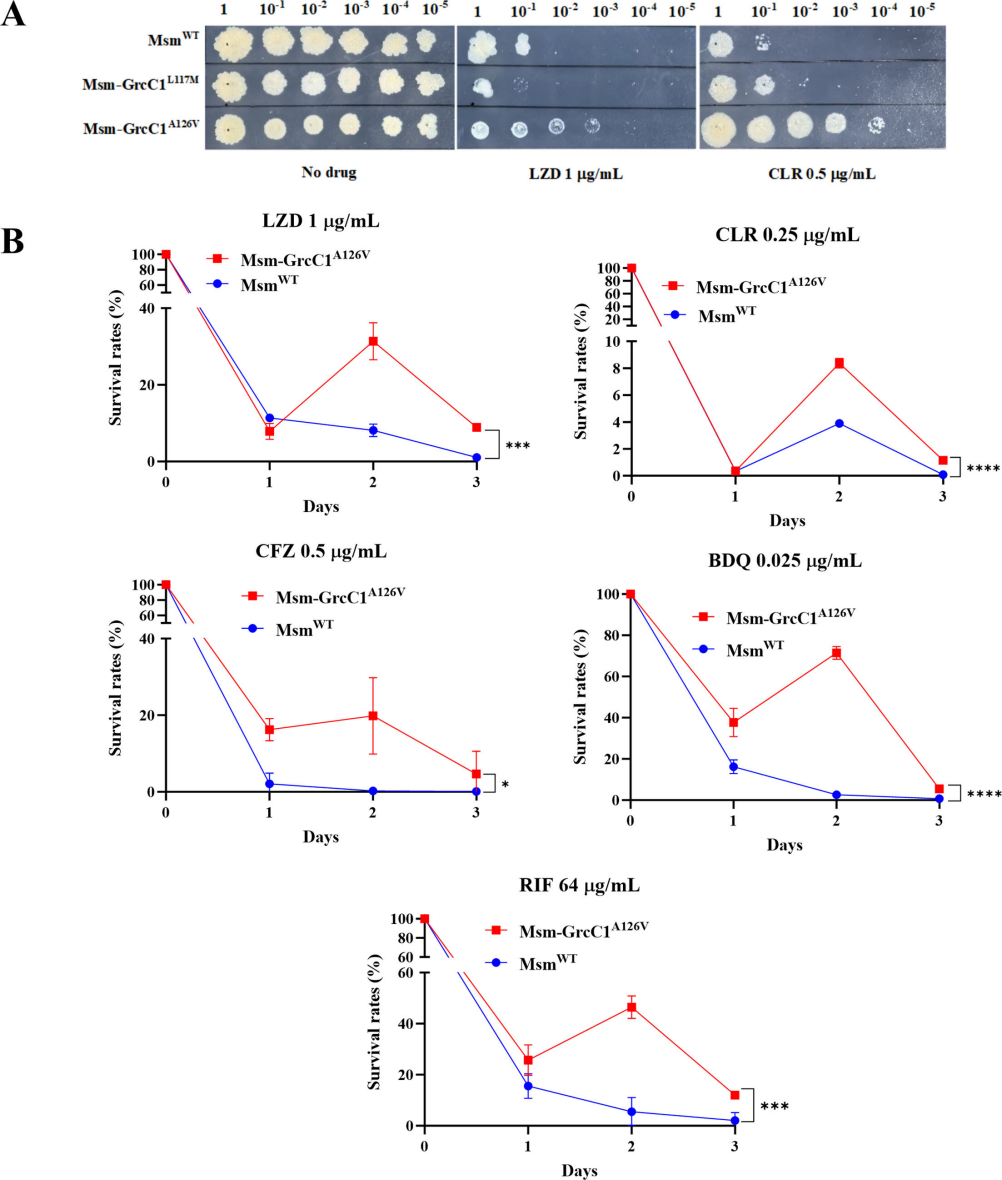
The A126V mutation at GrcC1 in Msm^WT^ conferred low-level resistance to various drugs. (**A**) Drug sensitivity of Msm^WT^, Msm-GrcC1^L117M^, and Msm-GrcC1^A126V^ to LZD and CLR on 7H10 agar. (**B**) Survival rates of Msm^WT^ and Msm-GrcC1^A126V^ following exposure to LZD, RIF, CFZ, and BDQ in the 7H9 medium. Data are presented as the mean ± SD with a sample size of 3. Statistical significance was determined using the AUC, followed by an unpaired *t*-test. Significance levels are denoted by ^*^ for *P* < 0.05, ^***^ for *P* < 0.001, and ^****^ for *P* < 0.0001.

DST of *grcC1*-edited *M. smegmatis* strains revealed that only the strain harboring the A126V mutation exhibited low-level resistance, primarily to LZD and CLR (Fig. 5A). When the survival rates of Msm^WT^ and Msm-GrcC1^A126V^ were assessed in the 7H9 medium after exposure to various drugs, significant resistance was observed only to LZD, CLR, RIF, CFZ, and bedaquiline (BDQ) (Fig. 5B). Notably, the drugs to which the edited strains exhibited significant resistance differed between 7H10 agar and 7H9 broth. The observed differences in drug resistance between 7H10 agar and 7H9 broth may be attributed to variations in bacterial growth, drug stability, and potential biofilm formation between solid and liquid media. Additionally, introducing the A126V mutation at GrcC1 in Msm^WT^ altered the colony morphology, resulting in smoother and more regular colonies compared to Msm^WT^ (Fig. S3). This observation suggests the potential impacts of the mutation on the cell envelope.

**Fig 6:**
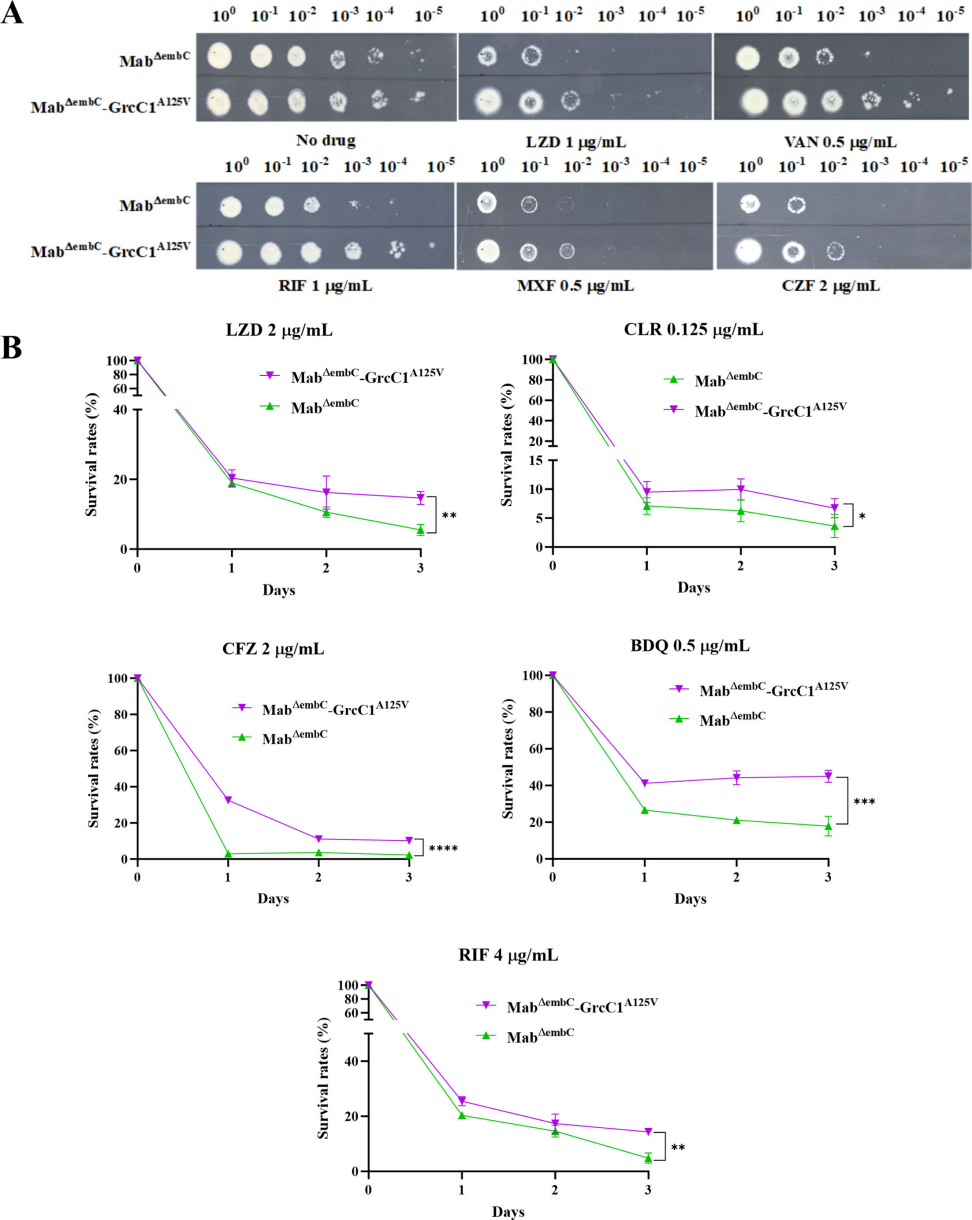
The A125V mutation at *grcC1* in Mab^ΔembC^ conferred low-level resistance to various drugs. (**A**) Drug sensitivity of Mab^ΔembC^ and Mab^ΔembC^–GrcC1^A125V^ to LZD, VAN, RIF, MXF, and CFZ on 7H10 agar. (**B**) Survival rates of Mab^ΔembC^ and Mab^ΔembC^–GrcC1^A125V^ following exposure to LZD, RIF, CFZ, and BDQ in 7H9 medium. Data are presented as the mean ± SD with a sample size of 3. Statistical significance was determined by calculating the AUC, followed by an unpaired *t* test, with ^*^ indicating *P* < 0.05, ^**^ indicating *P* < 0.01, ^***^ indicating *P* < 0.001, and ^****^ indicating *P* < 0.0001.

Similarly, the edited Mab^ΔembC^ strains displayed low-level resistance to a broader range of drugs on 7H10 agar, including LZD, VAN, RIF, MXF, and CFZ (Fig. 6A). In 7H9 medium, the Mab^ΔembC^–GrcC1^A125V^ also demonstrated significant resistance to LZD, CLR, RIF, CFZ, and BDQ (Fig. 6B). These findings confirm that the *grcC1*^A132V^ mutation plays a crucial role in conferring drug resistance in LZD-resistant AlMmr mutants and induces comparable resistance patterns across other mycobacterial species.

### Alterations in the cell wall permeability due to mutation, overexpression, or silencing of *grcC1*

**Fig 7:**
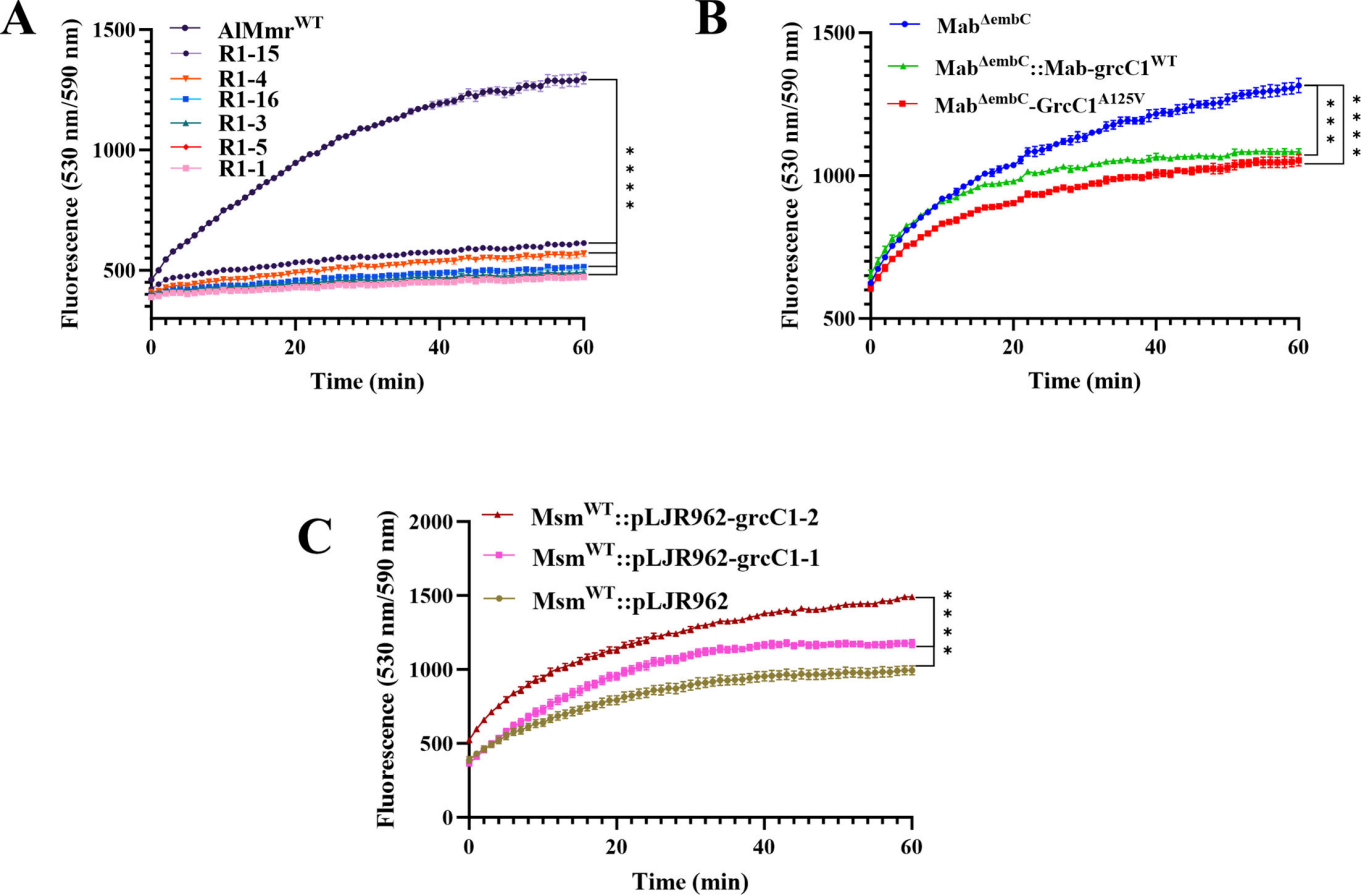
GrcC1 affected cell wall permeability in AlMmr, Mab^ΔembC^, and Msm^WT^. (**A**) Accumulation of EtBr in AlMmr and LZD-resistant mutants. (**B**) Accumulation of EtBr in Mab^ΔembC^, overexpression strains, and gene-edited strains. (**C**) Accumulation of EtBr in *grcC1*-silenced strains of Msm^WT^. Data are presented as the mean ± SD with a sample size of 3. Statistical significance was determined by one-way ANOVA, with ^***^ indicating *P* < 0.001 and ^****^ indicating *P* < 0.0001.

The ancestral function of GrcC1 is believed to initiate long-chain isoprenylated carriers, which is necessary for cell wall assembly (28). Given the importance of GGPP in the maintenance of membrane structure, we hypothesized that alterations in GrcC1 could impact cell wall permeability, potentially contributing to changes in drug susceptibility (14). To test this hypothesis, we assessed the cell wall permeability of our mutants and recombinant strains using the ethidium bromide (EtBr) uptake assay (19). The results showed a significant reduction in the cell wall permeability of LZD-resistant mutants of AlMmr compared to the parent strain AlMmr (Fig. 7A). Similarly, the Mab^ΔembC^ strain overexpressing *grcC1* from *M. abscessus* exhibited lower EtBr accumulation compared to Mab^ΔembC^, indicating reduced cell wall permeability (Fig. 7B). Furthermore, the introduction of the A125V mutation in *GrcC1* in Mab^ΔembC^ also resulted in reduced cell wall permeability, consistent with findings in resistant mutants (Fig. 7B). Conversely, silencing *grcC1* in Msm^WT^ significantly increased cell wall permeability (Fig. 7C). These observations imply that both overexpression and mutations in GrcC1 reduce cell wall permeability, contributing to drug resistance. The varied impact on cell wall permeability across different mycobacteria underscores the differential role of GrcC1 across these species, indicating a complex relationship between GrcC1 activity and cell wall integrity.

### The GrcC1^A132V^ mutation led to a reduction in the polarity of the glycolipid fraction in AlMmr, Msm^WT^, and Mab^ΔembC^ strains

To elucidate the underlying cause of the heightened cell wall permeability observed in LZD-resistant AlMmr mutants and the *grcC1*-edited Mab^ΔembC^ strain, we postulated that the *grcC1* mutation might alter the composition of cell wall lipids. To validate this hypothesis, lipid fractions were extracted from AlMmr and its resistant derivatives, alongside two *grcC1*-edited mycobacterial strains and their corresponding parental strains (Msm^WT^ and Mab^ΔembC^). These extracts underwent thin-layer chromatography (TLC) analysis for detailed examination.

**Fig 8:**
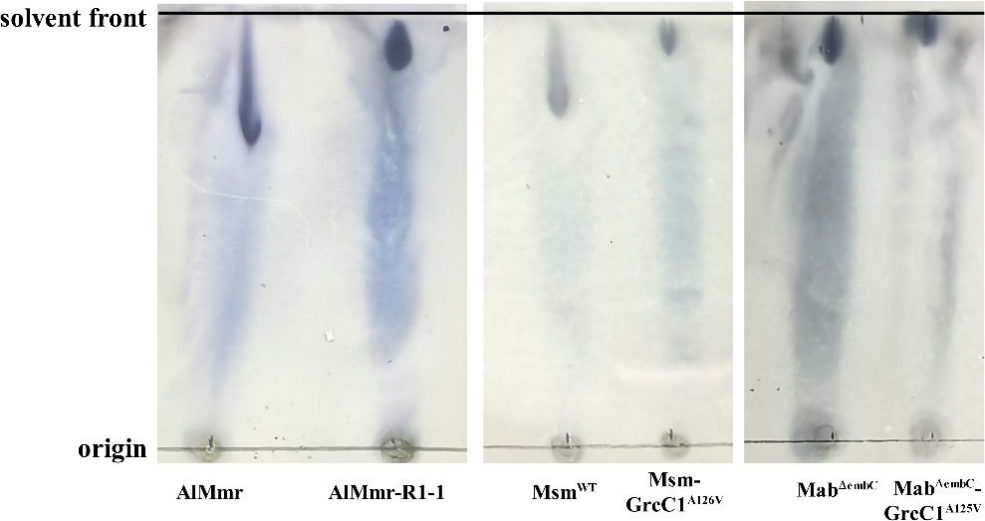
TLC analysis showing that GrcC1 mutation reduced the polarity of the glycolipid fraction in AlMmr, Msm^WT^, and Mab^ΔembC^. AlMmr-R1-1, an LZD-resistant AlMmr mutant haboring a GrcC1^A132V^ mutation; GrcC1^A126V^, homologous mutation of *M. marinum* GrcC1^A132V^ in *M. smegmatis*; GrcC1^A125V^, homologous mutation of *M. marinum* GrcC1^A132V^ in *M. abscessus*.

By examining the glycolipid profiles (Fig. 8), we observed that all *grcC1*-mutant strains exhibited a slightly higher retention factor (Rf) compared to their parental strains. Specifically, AlMmr had an Rf of 0.67, while its mutant R1-1 had an Rf of 0.88; Msm^WT^ showed an Rf of 0.77, and its mutant Msm–GrcC1^A126V^ had an Rf of 0.91; Mab^ΔembC^ displayed an Rf of 0.85, with its mutant Mab^ΔembC^–GrcC1^A125V^ showing an Rf of 0.91. These increases in Rf values suggested a decrease in polarity, likely due to an increase in the carbon chain length of the glycolipids (29). The reduction in glycolipid polarity implied an increase in hydrophobicity, which likely enhances the barrier function of the mycobacterial cell wall. These findings provide a mechanistic explanation for the reduced cell wall permeability associated with *grcC1* mutations, potentially contributing to drug resistance.

## DISCUSSION

Multidrug resistance complicates the treatment of TB and NTM infections, and emerging evidence of resistance mechanisms beyond known drug targets highlights the urgent need for new therapeutic strategies (1, 30). This study identifies a novel mutation in *grcC1*, a gene that has not been studied yet in association with drug resistance, which confers low-level resistance to multiple antimicrobial agents in *M. marinum*, *M. smegmatis*, and *M. abscessus*.

Overexpression is commonly employed to identify drug targets and resistance mechanisms, although it may have different outcomes across various genes and drugs (31). In our study, the overexpression of *MMAR_2832* and its homolog *Rv0890c* did not yield any resistance effects. This aligns with ongoing debates about the role of Rv0890c in LZD resistance (32, 33). Notably, Rv0890c acts as a regulatory factor that binds to DNA and RNA, potentially protecting bacterial DNA under stress conditions such as starvation and hypoxia (34). These interactions may not be triggered merely by overexpression, suggesting a more context-dependent role for *MMAR_2832* and *Rv0890c*. Additionally, the effect of overexpression varies in different strains; for instance, ClpC1 overexpression in mycobacteria showed varied resistance to cyclomarin A and ilamycins, contingent on the strain and mutation (35, 36). In our study, *grcC1* overexpression conferred resistance in a strain-specific manner in Msm^WT^, whereas in Mab^ΔembC^, overexpression of *grcC1* from *M. marinum* also conferred resistance, suggesting a more complex underlying mechanism. The challenge of generating constitutive *grcC1* overexpression strains in *M. marinum*—likely due to the gene’s essential nature—parallels difficulties seen with other essential genes, such as *yidC* in *M. tuberculosis*, where overexpression disrupted both growth and cell envelope integrity (37). These results indicate that *grcC1* plays a critical role in maintaining mycobacterial cell envelope integrity, influencing drug resistance through a complex regulatory mechanism.

Recent advancements in CRISPR screening, including CRISPRi and CRISPR-knockout techniques, have emerged as potent approaches for exploring genetic and chemical–genetic interactions in *M. tuberculosis* (38). For instance, a CRISPRi platform has been used to investigate the mechanism of various unknown genes and their involvement in drug resistance in *M. tuberculosis*, such as *tsnR*, where its silencing induces resistance to LZD (39). In our study, by employing these techniques, we found that *grcC1* is instrumental in Msm^WT^, Mab^ΔembC^, and *M. marinum*, yet it remains inconsequential in the Mab^WT^. Notably, disrupting *grcC1* in Mab^WT^ led to a growth defect, consistent with the genome-wide essentiality study using transposon mutagenesis, which showed that while *grcC1* can be disrupted, it causes growth defects (40). Inactivation of this protein affected growth and altered the MICs of various drugs against different NTM species, highlighting *grcC1* as a potential target for novel antimicrobial agents. Prior studies on LZD resistance mechanisms in *M. abscessus* primarily relied on indirect genetic validation through overexpression (41, 42). However, with advancements in CRISPR/Cpf1-assisted gene editing for rapidly growing mycobacteria, we directly confirmed the role of the *grcC1*^A132V^ in multidrug resistance in Msm^WT^ and Mab^ΔembC^ (26, 43).

Despite the established role of GrcC1 in cell wall synthesis, its association with bacterial drug resistance has not been previously explored (13, 44, 45). To the best of our knowledge, this study is the first to investigate the relationship between *grcC1* and drug resistance, thereby elucidating its potential impact on mycobacterial survival under drug pressure. Notably, alterations in lipid polarity and aberrant colony morphology exhibited by *grcC1*-deficient strains underscore the gene’s critical function in maintaining cell envelope homeostasis. Our data imply that genetic variations within *grcC1* might modulate enzymatic activities, potentially altering substrate specificity or chain lengths of lipids. These changes could exert indirect effects on cell wall architecture, influencing its composition and permeability, and ultimately impacting antimicrobial susceptibility.

Targeting prenyl diphosphate synthases like GrcC1 constitutes a potential approach in the development of novel antimicrobials. These enzymes play a crucial part in cell wall construction and are conserved among various pathogenic mycobacterial species, rendering them prime candidates for drug targeting. By inhibiting GrcC1, it is possible to undermine the structural integrity of the cell wall, thereby augmenting the effectiveness of current antimicrobial agents. This dual-action mechanism presents an advantageous therapeutic strategy.

In conclusion, our study identifies *grcC1* as a novel factor influencing both drug resistance and cell envelope integrity, providing new insights for understanding mycobacterial resistance mechanisms. The discovery of the A132V mutation in GrcC1 highlights its potential as a target for novel antimicrobial agents and underscores its significance as a diagnostic marker for resistant NTM infections. Further investigations should focus on elucidating the kinetic impact of *grcC1* mutations and exploring potential inhibitors targeting this enzyme. These efforts could pave the way for novel therapeutic strategies to combat resistant mycobacterial infections. Insights from our study will provide the base for developing more effective methodologies for diagnosing and countering resistant mycobacterial infections by designing new therapeutic agents.

## MATERIALS AND METHODS

### Plasmids, bacterial strains, reagents, and growth conditions

The bacterial strains and plasmids used in this study are listed in Table S1. *Escherichia coli Trans* T1 (TransGen Biotech) and derived strains were grown in lysogeny broth (LB) or agar at 37°C. All the mycobacterial strains were grown in Middlebrook 7H9 broth (BD), 7H10 agar (BD), or 7H11 agar (Acmec) supplemented with 10% OADC, 0.05% Tween 80, and 1% or 5% glycerol. The drug concentrations used for various bacteria were as follows: for *E. coli* and *M. smegmatis*, 50 µg/mL kanamycin (KAN, Solarbio), 50 µg/mL apramycin (APR, MeilunBio), and 30 µg/mL Zeocin (ZEO, Invivogen); for *M. marinum*, 50 µg/mL KAN and 50 µg/mL APR; for *M. abscessus*, 100 µg/mL KAN, 30 µg/mL ZEO, and 230 µg/mL APR. Anhydrotetracycline (aTc, Macklin) was used at 200 ng/mL for induced expression. AlMmr, *M. abscessus* GZ002, and *M. smegmatis* mc^2^155 were used as the wild-type strains (20, 22). *M. marinum* were cultured at 30°C, while *M. abscessus* and *M. smegmatis* were cultured at 37°C.

### Screening of LZD-resistant mutants without mutations in *rpLC* and *rrl* genes

AlMmr strains were cultured in 7H9 broth until the OD_600_ reached 1.0, then centrifuged at 4,000 rpm, and resuspended in one-tenth of the original culture volume with fresh 7H9 medium. The concentrated culture was plated onto 7H10 agar plates containing LZD at concentrations of 10, 20, and 40 µg/mL. Colonies that grew on the LZD-containing plates were screened for mutations in the *rplC* and *rrl* genes by colony PCR using primers Mm-rplCD-seq-F/R and Mm-rrl-seq-F/R, respectively (Table S2), and validated through Sanger sequencing. Colonies confirmed to lack mutations in these genes were further cultured in 7H9 broth to perform DST to LZD and confirm drug resistance.

### WGS analysis

The genomic DNAs of the parent AlMmr and the confirmed LZD-resistant mutants (without *rplC* and *rrl* mutations) were subjected to WGS by Shanghai Gene Optimal Biotechnology, China. Sequence reads were aligned with the genome of *M. marinum* strain M (NC_010612) and compared against that of the parent strain AlMmr. Mutations identified in the resistant mutants were validated through Sanger sequencing.

### Overexpression of *grcC1* and *MMAR_2832* in AlMmr, Msm^WT^, and Mab^ΔembC^

*grcC1*^WT^ and *MMAR_2832*^WT^, along with their mutant variants and homologous genes in *M. tuberculosis*, *M. smegmatis* and *M. abscessus*, were amplified from AlMmr, corresponding mutants, *M. tuberculosis* H37Ra, Msm^WT^, and Mab^WT^ by PCR using primers given in Table S2 and inserted into the pMV261A plasmid under the control of the *hsp60* promoter (Fig. S1). The resulting overexpression plasmids were then transformed into the AlMmr, Msm^WT^, and Mab^ΔembC^ via electroporation. Transformants were plated on 7H10 agar supplemented with APR. For AlMmr and Msm^WT^ strains, 50 µg/mL of APR was used, while 230 µg/mL was used for Mab^ΔembC^. Positive recombinant strains were identified by PCR using primers given in Table S2. The drug susceptibilities of the recombinant strains were determined as described above.

### CRISPRi-assisted *grcC1* gene silencing in Msm^WT^, Mab^WT^, Mab^ΔembC^, and Mmr^WT^

We utilized the dCas9^sth1^-based CRISPRi system for targeted gene silencing (23). Small guide RNAs (sgRNAs) of 20 base pairs were designed to target the coding regions of *grcC1* on the sense strands in different *Mycobacterium* species. The oligonucleotides were annealed and then ligated with linearized pLJR962 (Addgene) (23). Plasmids harboring sgRNAs, along with the empty vector pLJR962 as a control, were introduced into *M. marinum*, Mab^WT^, Mab^ΔembC^, and Msm^WT^ cells via electroporation as described above. The gene-silenced strains and the control strain carrying the empty vector were cultured, and the efficacy of gene silencing was assessed.

For assessment, tenfold serial dilutions of *M. marinum*, Mab^WT^, Mab^ΔembC^, and Msm^WT^, the control strains carrying the empty pLJR962 vector, and the gene-silenced strains were prepared after reaching an OD_600_ of approximately 0.9. Subsequently, 1 µL of each dilution was spotted on plates containing various drugs at a concentration of 1/4 MIC with different concentrations of aTc. aTc was used at concentrations of 50, 100, and 200 ng/mL for *M. marinum*; 100, 200, and 400 ng/mL for *M. abscessus*; and 25, 50, and 100 ng/mL for *M. smegmatis*. Control plates were supplemented with only aTc. The experiment was performed in triplicate and repeated twice.

### CRISPR/Cpf1-assisted *grcC1* knockout in Mab^WT^

To generate the *grcC1* knockout strain in Mab^WT^, we applied the CRISPR/Cpf1-assisted NHEJ strategy as previously described (24). The pNHEJ-Cpf1 plasmid was first electroporated into Mab^WT^. Competent cells of recombinant strain were prepared by washing it with ice-cold 10% glycerol. The crRNAs were designed using https://chopchop.cbu.uib.no/ (Table S3). Oligonucleotides for crRNA expression were ligated into the pCR-Zeo vector. These constructed plasmids were then electroporated into the competent cells of Mab::pNHEJ-Cpf1. Transformants were plated on 7H11 agar supplemented with KAN, ZEO, and aTc and incubated at 30℃. Positive colonies were identified by PCR and Sanger sequencing. The drug susceptibilities of the recombinant strains were determined as described above.

### CRISPR/Cpf1-assisted gene editing in Msm^WT^ and Mab^ΔembC^

CRISPR/Cpf1-assisted recombineering has been employed for gene editing in both *M. abscessus* and *M. smegmatis* (26, 43). Initially, the pJV53-Cpf1 plasmid was electroporated into Mab^ΔembC^ and Msm^WT^ strains, resulting in the construction of Mab^ΔembC^::pJV53-Cpf1 and Msm^WT^::pJV53-Cpf1, respectively. Subsequently, crRNAs were designed to target specific 25 nucleotide sequences downstream of the 5′-YTN-3′ motif near the target site in *grcC1* (Table S3). Oligonucleotides for crRNA expression were ligated into the pCR-Zeo vector. These plasmids were then electroporated into the respective acetamide-induced cells of Mab^ΔembC^::pJV53-Cpf1 or Msm^WT^::pJV53-Cpf1. Transformants were plated on 7H11 agar plates supplemented with KAN, ZEO, and aTc and incubated at 30℃. Individual colonies were picked and screened to confirm successful editing at the target gene site via PCR and Sanger sequencing (primers listed in Table S2). The drug susceptibilities of the edited strains were determined using the previously described method.

### DST

The MICs against AlMmr, derived resistant strains, and overexpressing strains were determined using a cost-effective *in vitro* assay as previously described (22). The MIC_lux_ was defined as the minimum concentration inhibiting > 90% RLUs compared to untreated controls (22). The MICs against *M. abscessus* and *M. smegmatis* were determined using the microtiter broth dilution method (19), by incubating at 37°C for 3 days, with the MIC defined as the lowest concentration resulting in clear turbidity compared to the untreated control cultures.

Additionally, DST on agar plates was performed as follows: tested strains were cultured to the exponential phase and adjusted using 7H9 broth until the OD_600_ reached approximately 0.9. Subsequently, the cultures were standardized using 7H9 broth, followed by a tenfold serial dilution. From each dilution, 1 µL of the bacterial suspension was spotted onto 7H10 agar plates containing different drugs at a concentration of 1/2 or 1 × the MIC. The experiments were conducted in triplicate and repeated twice.

### Growth curves

Mab^WT^, Mab^ΔgrcC1^, and Mab^ΔgrcC1^::cMab strains were inoculated into 7H9 medium with KAN at a final concentration 100 µg/mL, which was added to the broth containing the complemented strain. Cultures were grown until the OD_600_ reached approximately 0.5 ~ 0.8. Subsequently, the bacterial cultures were adjusted to an OD_600_ of 0.5 using 7H9 medium to ensure consistent starting concentrations for all strains. For each strain, 250 µL of the adjusted culture was inoculated into flasks containing 50 mL of 7H9 medium with sterilized glass beads. Triplicates were set up for each strain, and the flasks were cultured at 37°C with shaking at 200 rpm.

Sampling starting from 0 hours post-inoculation, with culture aliquots of 1.6 mL collected every 6 hours for OD_600_ measurements using a UV spectrophotometer (AOE). This process continued until 72 hours post-inoculation. Growth curves were generated by plotting the OD_600_ values against time in GraphPad Prism 8. Subsequently, AUC values were computed, and statistical analyses were performed utilizing one-way ANOVA to compare the groups.

### Survival rates

To assess the survival rate under drug treatment, an integrative plasmid carrying the *luxCDEAB* operon was introduced into both edited and parental strains to facilitate autonomous bioluminescence (46, 47). The strains were cultured, adjusted to an OD_600_ of ~0.5, and diluted 1,000-fold to prepare the inoculum. Following drug exposure, RLUs were measured at designated time points. Survival rates were calculated by normalizing the RLUs of each edited strain to that of its parental strain at the same time point and expressing the result as a percentage. Time-kill curves were generated using GraphPad Prism 8, and the AUC was calculated for statistical comparisons. An unpaired *t*-test was used to determine statistical significance.

### Detection of cell wall permeability

The EtBr uptake assay was performed as described previously (19). Tested strains were cultured until OD_600_ reached 0.6 ~ 1.0. Cells were washed twice with PBS containing 0.05% Tween-80 and then adjusted to a final OD_600_ of 0.5. The EtBr working solution was prepared with 0.08% glucose and 4 µg/mL EtBr. For each strain, 100 µL of the cell suspension was added to six wells in a white 96-well plate, with three wells each for the experimental and control groups. Fluorescence was measured using a FlexStation 3 microplate reader (Molecular Devices) set at 530 nm excitation and 590 nm emission, recorded every minute for 60 minutes after EtBr addition. The data were corrected by subtracting the no-cell control fluorescence from the experimental values and then analyzed using GraphPad Prism 8. Results are presented as the mean ± SD, with *n* = 3 for each group. An unpaired *t*-test was applied to determine statistical significance, with *P* < 0.05 considered significant.

### Lipid extraction

Bacterial cultures were grown to an OD_600_ of approximately 1.0 and harvested by centrifugation at 6,000 rpm. Supernatants were discarded, and the cell pellets were weighed to ensure equal sample mass. Pellets were resuspended in 50 mL of a methanol:chloroform:0.3% NaCl solution (9:10:3, vol/vol/vol) and shaken at room temperature for 4 hours. Insoluble impurities were removed by filtering through a magnesium silicate column (Green Mall). The column was washed twice with 10 mL of methanol:chloroform:0.3% NaCl (5:10:4, vol/vol/vol). The filtered solution was subsequently mixed with 20 mL of chloroform:0.3% NaCl (1:1, vol/vol) and shaken for 1 hour. After phase separation, the lower organic phase containing the lipids was carefully collected. Total cell wall lipids were obtained by drying the organic phase under reduced pressure using rotary evaporation.

### TLC

The extracted lipids were dissolved in 500 µL of a chloroform:methanol solution (2:1, vol/vol). A 1.5 µL aliquot of each sample was spotted onto the GF254 silica gel plates (Qingdao Haiyang Chemical) and developed in a solvent system of chloroform:acetone:methanol:water (50:60:2.5:3) (48). After air-drying, the plates were sprayed with an anisaldehyde reagent (0.5 mL p-anisaldehyde, 10 mL acetic acid, 85 mL methanol, and 4.5 mL sulfuric acid) and charred to visualize the spots (49). The distances traveled by each lipid sample and the solvent front were measured, and Rf values were calculated using the formula:


$$Rf=\frac{\text{ Distance traveled by the sample }}{\text{ Distance traveled by the solvent front }}.$$

## SUPPLEMENTAL MATERIAL

Supplemental material spectrum.02289-24-s0001.docx Fig. S1 to S3; Tables S1 to S3.

ASM does not own the copyrights to Supplemental Material that may be linked to, or accessed through, an article. The authors have granted ASM a non-exclusive, world-wide license to publish the Supplemental Material files. Please contact the corresponding author directly for reuse.
