# Supplementary material for: GrcC1 mediates low-level resistance to multiple drugs in M. marinum, M. abscessus, and M. smegmatis
Source: Microbiol Spectr. 2025 Feb 26;13(4):e02289-24. doi: 10.1128/spectrum.02289-24 (PMC11960048; doi:10.1128/spectrum.02289-24)
Supplement: Supplemental material — Fig. S1 to S3; Tables S1 to S3. [file spectrum.02289-24-s0001.docx]

**Supplementary Materials**

**for**

**GrcC1 mediates low-level resistance to multiple drugs in *M. marinum*, *M. abscessus* and *M. smegmatis***


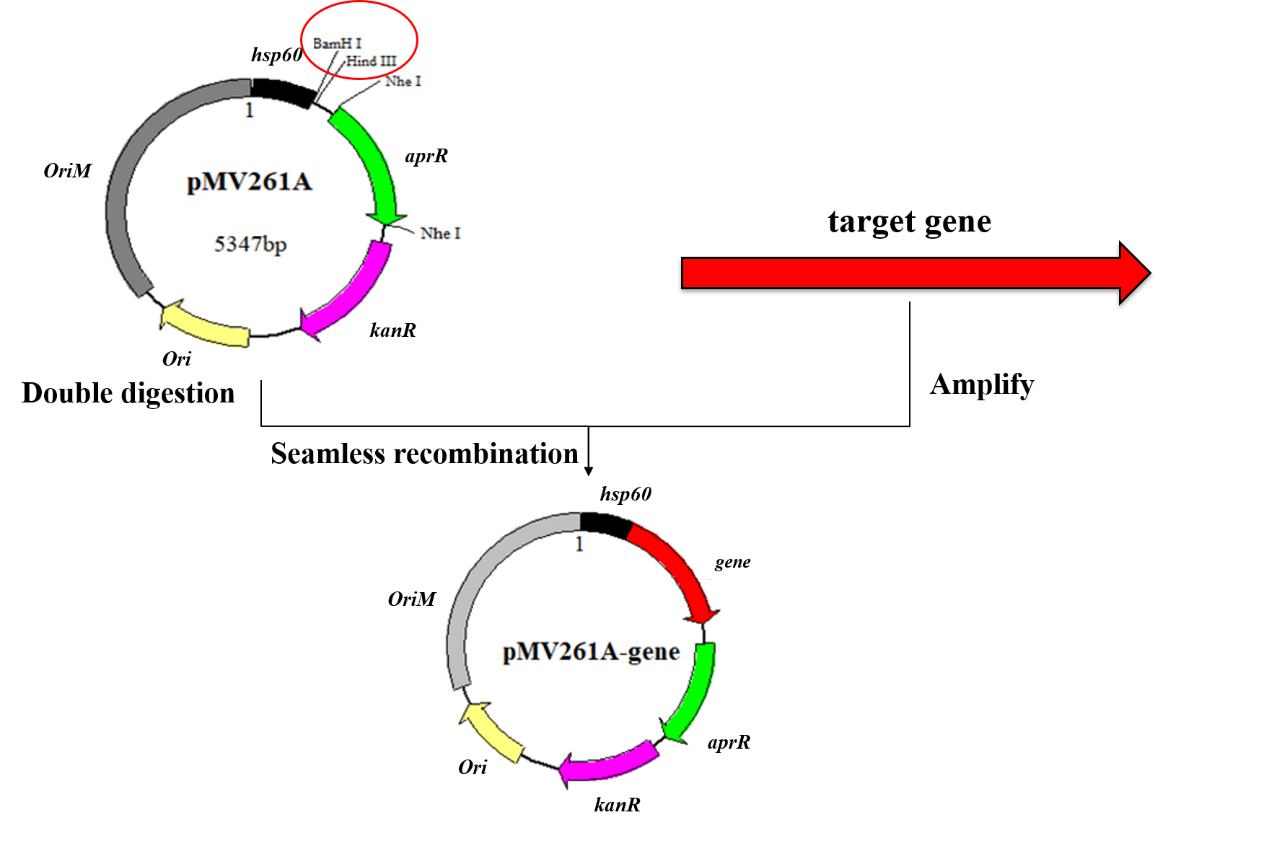


**Fig S1** Schematic diagram of constructing the overexpression plasmids. *hsp60*, a mycobacterial strong promoter; *Ori,* origin of replication in *Escherichia coli*; *OriM*, origin of replication in Mycobacteria; *aprR*, aparamycin resistant gene; *kanR*, kanamycin resistant gene.


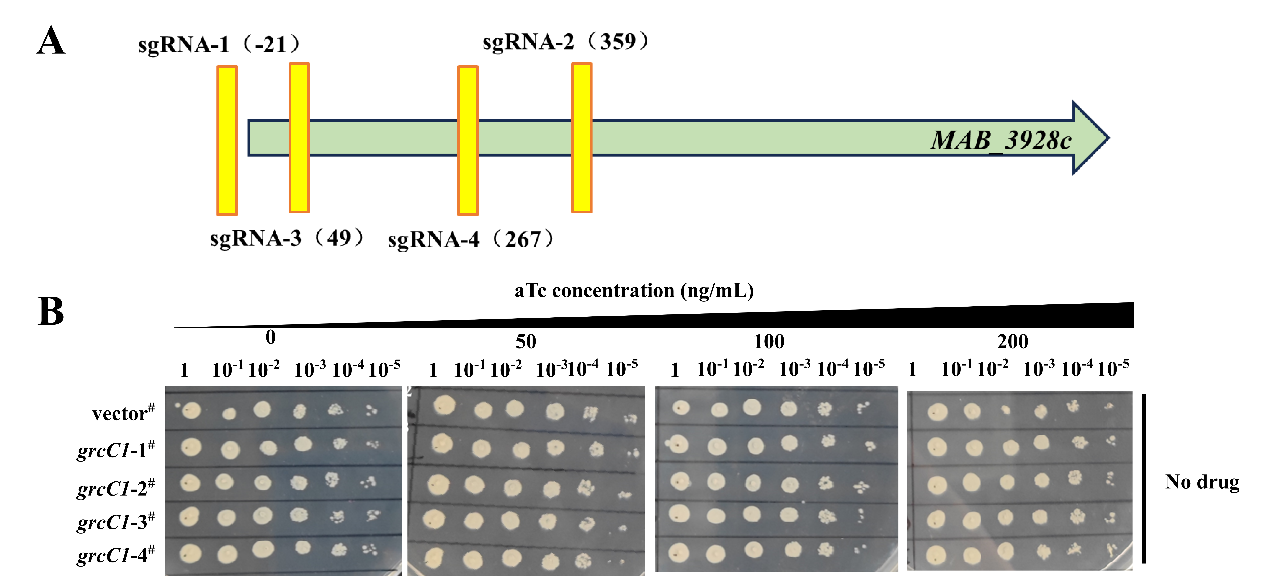


**Fig S2** Silencing *grcC1* had no effect on growth in Mab^WT^. (A) Schematic diagram showing the locations of sgRNAs designed for silencing *grcC1* in Mab^WT^. The numbers in brackets represent the midpoint positions of the sgRNAs within the gene. (B) Spot assay showing different dilutions of Mab^WT^ and derived silenced strains on 7H11 plates supplemented with different concentrations of aTc. vector, control strain harboring the empty vector pLJR962; aTc, inducer for the expression of sgRNAs.


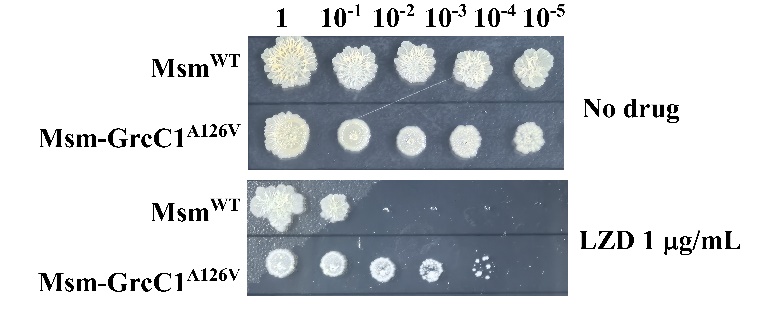


**Fig S3** Spot assay showed gene-edited strain Msm-GrcC1^A126V^ became smoother and more regular compared to Msm^WT^.

**Table S1** Strains used in this study

| **Strains** | **Description** | **Source or reference** |
| --- | --- | --- |
| AlMmr | Selectable marker-free autoluminescent Mmr | (1) |
| Mmr^WT^ | Mmr strain M | Lab stock |
| Mab^WT^ | Mab strain GZ002 | (2) |
| Msm^WT^ | Msm strain mc^2^155 | Lab stock |
| Mab^ΔembC^ | *embC* gene knockout strain of Mab GZ002 | (3) |
| AlMmr::MM2832^WT^ | AlMmr carrying plasmid pMV261A-MMAR_2832 ^WT^ | This study |
| AlMmr::MM2832^MU^ | AlMmr carrying plasmid pMV261A-MMAR_2832 ^WT^ | This study |
| Msm::MM2832^WT^ | Msm^WT^ carrying plasmid pMV261A-MMAR_2832 ^WT^ | This study |
| Msm::MM2832^MU^ | Msm^WT^ carrying plasmid pMV261A-MMAR_2832 ^WT^ | This study |
| Mab^ΔembC^::MM2832^WT^ | Mab^ΔembC^ carrying plasmid pMV261A-MMAR_2832 ^WT^ | This study |
| Mab^ΔembC^::MM2832^MU^ | Mab^ΔembC^ carrying plasmid pMV261A-MMAR_2832 ^WT^ | This study |
| Msm::Mmr-grcC1^WT^ | Msm^WT^ carrying plasmid pMV261A-Mm-grcC1^WT^ | This study |
| Msm::Mmr-grcC1^MU^ | Msm^WT^ carrying plasmid pMV261A-Mm-grcC1^MU^ | This study |
| Msm::Msm-grcC1 | Msm^WT^ carrying plasmid pMV261A-Msm-grcC1^WT^ | This study |
| Msm::Mab-grcC1 | Msm^WT^ carrying plasmid pMV261A-Mab-grcC1^WT^ | This study |
| Msm::Mtb-grcC1 | Msm^WT^ carrying plasmid pMV261A- Mtb-grcC1^WT^ | This study |
| Mab^ΔembC^::Mmr-grcC1^WT^ | Mab^ΔembC^ carrying plasmid pMV261A-Mm-grcC1^WT^ | This study |
| Mab^ΔembC^::Mmr-grcC1^MU^ | Mab^ΔembC^ carrying plasmid pMV261A-Mm-grcC1^MU^ | This study |
| Mab^ΔembC^::Msm-grcC1 | Mab^ΔembC^ carrying plasmid pMV261A-Msm-grcC1^WT^ | This study |
| Mab^ΔembC^::Mab-grcC1 | Mab^ΔembC^ carrying plasmid pMV261A-Mab-grcC1^WT^ | This study |
| Mab^ΔembC^::Mtb-grcC1 | Mab^ΔembC^ carrying plasmid pMV261A-Mtb-grcC1^WT^ | This study |
| Mmr::pLJR962 | Mmr^WT^ carrying plasmid pLJR962 | This study |
| Mmr-sgRNA-1 | Mmr^WT^ carrying plasmid pLJR962-grcC1-sgRNA-1 | This study |
| Mmr-sgRNA-2 | Mmr^WT^ carrying plasmid pLJR962-grcC1-sgRNA-2 | This study |
| Msm::pLJR962 | Msm^WT^ carrying plasmid pLJR962 | (4) |
| Msm-sgRNA-1 | Msm^WT^ carrying plasmid pLJR962-grcC1-sgRNA-1 | This study |
| Msm-sgRNA-2 | Msm^WT^ carrying plasmid pLJR962-grcC1-sgRNA-2 | This study |
| Mab^ΔembC^::pLJR962 | Mab^ΔembC^ carrying plasmid pLJR962 | This study |
| Mab^ΔembC^-sgRNA-1 | Mab^ΔembC^ carrying plasmid pLJR962-grcC1-sgRNA-1 | This study |
| Mab^ΔembC^-sgRNA-2 | Mab^ΔembC^ carrying plasmid pLJR962-grcC1-sgRNA-2 | This study |
| Mab::pLJR962 | Mab^WT^ carrying plasmid pLJR962 | This study |
| Mab-sgRNA-1 | Mab^WT^ carrying plasmid pLJR962-grcC1-sgRNA-1 | This study |
| Mab-sgRNA-2 | Mab^WT^ carrying plasmid pLJR962-grcC1-sgRNA-2 | This study |
| Mab-sgRNA-3 | Mab^WT^ carrying plasmid pLJR962-grcC1-sgRNA-3 | This study |
| Mab-sgRNA-4 | Mab^WT^ carrying plasmid pLJR962-grcC1-sgRNA-4 | This study |
| Mab::pNHEJ-Cpf1 | Mab^WT^ carrying plasmid pNHEJ-Cpf1 | (5) |
| Mab^ΔgrcC1^ | *grcC1* gene disruption strain of Mab GZ002 | This study |
| Mab^ΔgrcC1::CMab^ | Mab^ΔgrcC1^ complemented with pMV261A-Mab-grcC1^WT^ | This study |
| Msm::pJV53-Cpf1 | Msm^WT^ carrying plasmid pJV53-Cpf1 | This study |
| Msm-grcC1^L117M^ | Msm^WT^ in which *grcC1*^WT^ was edited into *grcC1*^L117M^ | This study |
| Msm-grcC1^A126V^ | Msm^WT^ in which *grcC1*^WT^ was edited into *grcC1*^A126V^ | This study |
| Mab^ΔembC^::pJV53-Cpf1 | Mab^ΔembC^ carrying plasmid pJV53-Cpf1 | (6) |
| Mab^ΔembC^-grcC1^A126V^ | Mab^ΔembC^ in which *grcC1*^WT^ was edited into *grcC1*^A126V^ | This study |

**Table S2** Primers used in this study

| **Primers** | **Primer sequences (5’-3’)** | **Purpose** |
| --- | --- | --- |
| Mm-rplCD-seq-F | cctacgaccacgaggctattga | Used for *rplC* and *rrl* sequencing in Mmr, Msm, and Mab. |
| Mm-rplCD-seq-R | tgttcaccgatgcgaccttg |  |
| Mm-rrl-seq-F | acaacaacaaagcaagccagaca |  |
| Mm-rrl-seq-R | gaccaacggcggttacgg |  |
| Mab-rplCD-seq-F | cgagatgcgtacccacaagc |  |
| Mab-rplCD-seq-R | tggcgatcttgatctgcgtct |  |
| Mm-rplCD-seq-F | cctacgaccacgaggctattga |  |
| Mm-rplCD-seq-R | tgttcaccgatgcgaccttg |  |
| Mm-rrl-seq-F | acaacaacaaagcaagccagaca |  |
| Mm-rrl-seq-R | gaccaacggcggttacgg |  |
| Mab-rplCD-seq-F | cgagatgcgtacccacaagc |  |
| Mab-rplCD-seq-R | tggcgatcttgatctgcgtct |  |
| Mab-rrl-seq-F | gactgtcataagaattgaaacgctggc |  |
| Mab-rrl-seq-R | cggtgtcctactcttccgttcc |  |
| Msm-rplCD-seq-F | cggtgtgcaggtggttgtggac |  |
| Msm-rplCD-seq-R | gttcaccgaatcgaccttgacg |  |
| Msm-rrlA-seq-F | gtgggaggcgtgttgttgcc |  |
| Msm-rrlA-seq-R | cttttccacccggaagggta |  |
| Msm-rrlB-seq-F | gtgtggtgtttgatttgt |  |
| Msm-rrlB-seq-R | tttccacccggatgggtagta |  |
| Mm-grcC1-seq-F | cgccgaatccgctcatgc | Used for *grcC1* sequencing in Mmr. |
| Mm-grcC1-seq-R | tgtcgctgttgaagtagacatagacg |  |
| Mm2382-seq-F | cagcacacggcccaacgc | Used for *MMAR_2832* sequencing in Mmr. |
| Mm2382-seq-R | cgcctcgcttggcaaaatg |  |
| Mtb-grcC1-CZ-F | agacaattgcggatccgtgaggactccggcgacg | To amplify *grcC1* genes from Mtb for cloning into the overexpression vector pMV261A. |
| Mtb-grcC1-CZ-R | cgacatcgataagcttctagccgtgccggctca |  |
| Mm-grcC1-CZ-F | agacaattgcggatccatgagaacctcggcg | To amplify *grcC1* genes from Mmr for cloning into the overexpression vector pMV261A. |
| Mm-grcC1-CZ-R | cgacatcgataagcttctacccgtgccggttgaccgt |  |
| Mab-grcC1-CZ-F | cgacatcgataagcttttatccgtggcggcgc | To amplify *grcC1* genes from Mab for cloning into the overexpression vector pMV261A. |
| Mab-grcC1-CZ-R | agacaattgcggatccgtggccggagttgacct |  |
| Msm-grcC1-CZ-F | agacaattgcggatccgtggtggcaggcgttg | To amplify *grcC1* genes from Msm for cloning into the overexpression vector pMV261A. |
| Msm-grcC1-CZ-R | cgacatcgataagctttcagccttcccggctgat |  |
| Mm2832-CZ-F | ggccaagacaattgcggatccttgaattcgcgactggtgacg | To amplify *MMAR_2832* genes from Mmr for cloning into the overexpression vector pMV261A. |
| Mm2832-CZ-R | tacgtcgacatcgataagctttcacccgccataccctcg |  |

**Table S3** The crRNAs, sgRNAs and oligonucleotides used for gene silencing, knockout, and editing.

| **crRNAs, sgRNAs and oligonucleotides** | **Description** |
| --- | --- |
| ccacttgttcgccgtcggcg | sgRNA-1 for scilencing *grcC1* in Mmr |
| tccccagtatgttggtcgga | sgRNA-2 for scilencing *grcC1* in Mmr |
| tactcactctgccaacatac | sgRNA-1 for scilencing *grcC1* in Msm |
| ggcgaacaggtagtcgccgg | sgRNA-2 for scilencing *grcC1* in Msm |
| tactcaccggtccaacatac | sgRNA-1 for scilencing *grcC1* in Mab |
| ggccagcagatagtccccgg | sgRNA-2 for scilencing *grcC1* in Mab |
| attcgccgccaaggtgcgcg | sgRNA-3 for scilencing *grcC1* in Mab |
| gacgacgtcatggacgaggc | sgRNA-4 for scilencing *grcC1* in Mab |
| gatctgatcgcagcgcgac | crRNA for disruption of *grcC1* in Mab |
| gccggcgactacctgttcgccac | crRNA targeting the L117 of *grcC1* in Msm |
| gacgccgtggcgaacaggtagtcgccggcCaTgatcgcgatgttgttgccccagcgtgc | oligonucleotides template for editing into L117M in *grcC1* of Msm |
| gccacggcgtcgcgactcgtgtc | crRNA targeting the A126 of *grcC1* in Msm |
| gggacccagacgggacacgagtcgcgacAccgtggcgaacaggtagtcgccggcgagga | oligonucleotides template for editing into A126V in *grcC1* of Msm |
| tctgctggccacggcctcgcggttg | crRNA targeting the A125 of *grcC1* in Mab |
| tggggcccagccgcgacaccaaccgcgaTAccgtggccagcagatagtccccggccaga | oligonucleotides template for editing into A125V in *grcC1* of Mab |

**References**

1. Liu Y, Gao YM, Liu JX, Tan YJ, Liu ZY, Chhotaray C, Jiang HF, Lu ZL, Chiwala G, Wang S, Makafe G, Islam MM, Hameed HMA, Cai XS, Wang CW, Li XJ, Tan SY, Zhang TY. 2019. The compound TB47 is highly bactericidal against *Mycobacterium ulcerans* in a Buruli ulcer mouse model. Nat Commun 10:524. https://doi.org/10.1038/s41467-019-08464-y

2. Chhotaray C, Wang S, Tan Y, Ali A, Shehroz M, Fang C, Liu Y, Lu Z, Cai X, Hameed HMA, Islam MM, Surineni G, Tan S, Liu J, Zhang T. 2020. Comparative analysis of whole-genome and methylome profiles of a smooth and a rough *Mycobacterium abscessus* clinical strain. G3 (Bethesda, Md) 10:13-22. https://doi.org/10.1534/g3.119.400737

3. Wang S, Cai X, Yu W, Zeng S, Zhang J, Guo L, Gao Y, Lu Z, Hameed HMA, Fang C, Tian X, Yusuf B, Chhotaray C, Alam MDS, Zhang B, Ge H, Maslov DA, Cook GM, Peng J, Lin Y, Zhong N, Zhang G, Zhang T. 2022. Arabinosyltransferase C mediates multiple drugs intrinsic resistance by altering cell envelope permeability in *Mycobacterium abscessus*. Microbiol Spectr 10:e0276321. https://doi.org/10.1128/spectrum.02763-21

4. Han X, Gao Y, Zhou B, Hameed HMA, Fang C, Ju Y, He J, Fang X, Liu Z, Yu W, Xiong X, Zhong N, Zhang T. 2024. Indole propionic acid disturbs the normal function of tryptophanyl-tRNA synthetase in *Mycobacterium tuberculosis*. ACS Infect Dis 10:1201-1211. https://doi.org/10.1021/acsinfecdis.3c00585

5. Zeng S, Ju Y, Alam MS, Lu Z, Hameed HMA, Li L, Tian X, Fang C, Fang X, Ding J, Wang X, Hu J, Wang S, Zhang T. 2024. Disrupting homologous recombination or single-strand annealing significantly hinders CRISPR-Cas12a-assisted nonhomologous end-joining gene editing efficiency in *Mycobacterium abscessus*. bioRxiv. https://doi.org/10.1101/2024.01.29.577284

6. He J, Gao Y, Wang J, Hameed H. M. A, Wang S, Fang C, Tian X, Zhang J, Han X, Ju Y, Tan Y, Ma J, Ju J, Hu J, Liu J, T. Z. 2024. EmbB and EmbC regulate the sensitivity of *Mycobacterium abscessus* to echinomycin. mLife 3:459-470. 10.1002/mlf2.12139
